# Bacterial loads and antibiotic resistance profile of bacteria isolated from the most popular street food (*Phuchka*) in Bangladesh

**DOI:** 10.5455/javar.2021.h523

**Published:** 2021-07-03

**Authors:** Mahadi Hasan, Farzana Siddika, Md. Arefin Kallol, Najibullah Sheikh, Muhammad Tofazzal Hossain, MD. Mahmudul Alam, Marzia Rahman

**Affiliations:** 1 Department of Microbiology and Hygiene, Bangladesh Agricultural University, Mymensingh-2202, Bangladesh; 2 Department of Surgery and Obstetrics, Bangladesh Agricultural University, Mymensingh-2202, Bangladesh

**Keywords:** Street food, *Phuchka*, Bacterial load, Antibiotic resistance

## Abstract

**Objective:**

*Phuchka* is one of the most common street foods in Bangladesh. It is served with salad, sweet and sour tamarind dispersed water, and minced eggs as topping at places where people usually gather. This makes these foods susceptible to bacterial contamination. Therefore, assessing the bacterial load and antimicrobial profile of organisms isolated from *phuchka* and other foodstuffs served with it was the focus of this study.

**Materials and Methods:**

Bacterial isolates were isolated and identified from the samples after the bacterial loads were assessed as total viable count (TVC), total coliform count (TCC), and total staphylococcal count (TSC). The antibiotic resistance profile of the isolates was obtained using the disk diffusion method. Molecular detection of *Escherichia coli* isolates and the presence of gene responsible for tetracycline resistance was confirmed by polymerase chain reaction.

**Results:**

According to the recommendations, the TVC value of 70% *phuchka* and egg samples was safe, whereas TSC value illustrated that 80% of both *phuchka* and egg samples were at safety level. For the TCC value, 80% egg and 70% *phuchka* samples were found to be safe for consumption. Among all the samples, the microbial loads of the vendors’ hand wash were least in the safety level. Antibiotic sensitivity tests revealed that both *Staphylococcus* spp. and *E. coli* isolates were sensitive to gentamicin and ciprofloxacin but showed resistance to ampicillin.

**Conclusion:**

The data of this study indicate that *phuchka* can pose a public health problem as foodborne bacterial isolates which are antibiotic-resistant are found in it.

## Introduction

*Phuchka* is a popular street food in Bangladesh found all over the country. It is a crispy hollow morsel filled with spiced ingredients, such as boiled, mashed potato and matar, salad, minced egg, and green chili, and is served with sweet and sour tamarind sauce. *Phuchka* is prepared in large volumes by vendors and consumed by customers because of its easy preparation, affordable price, and superior taste [1].

Street food business guarantees economic security of many low-income populations and ensures their livelihood [2]. Often, most street food vendors are poor and have little or no knowledge about foods safely, risk of contamination, the importance of sanitation, and hygiene. Because of these factors, street foods remain a significant origin of public health risk factors. According to a Food and Agricultural Organization report, the number of people who eat street food every day is around 2.5 billion [3].

Opening a street food business does not require any training and certification; there is a very high chance of violating food safety and hygiene rules. Each year in Bangladesh, the number of people who suffer from foodborne illness is nearly 30 million. Street foods have a fair share of this number. Bacterial and fungal contamination of foods usually occurs from machines, utensils, water, etc., during harvesting and processing [4–6].

Outbreaks caused by foodborne diseases are considered a major health risk throughout the world, and in developing countries like Bangladesh, the risk is higher [7–9]. Compelled by modern urbanization, many people living in the city eat street foods to meet their daily nutritional requirements. Contaminated foods may lead to severe health risks to consumers [6]. The data obtained from previous studies show that street foods can spread microorganisms, including *Bacillus*, *Staphylococcus*, *Escherichia coli*, *Clostridium*, *Vibrio*, *Campylobacter*, *Listeria*, and *Salmonella*, to a level which can cause diseases in humans [10,11].

The microbial assessments of chicken roll, spicy puffed rice, hog plum, and pickles were reported in Bangladesh [8,10,12], and a study on *phuchka* sellers’ hand wash was carried out in Dhaka [13]. No survey on the microbial quality of *phuchka*, its egg toppings, and salad has been conducted before our study to the best of our understanding. Because *phuchka* is the most common street food and consumed all over the country, it is important to report the microbiological quality of all components of *phuchka* to weigh the risk factors associated with it. 

## Materials and Methods

### Ethical statement

All participants in the study were informed about the study and verbal consent was taken from them.

### Selection of site and description of the food samples

To determine the bacteriological quality of *phuchka* and associated foodstuff, samples were taken from distinct sites of the Bangladesh Agricultural University campus and some densely populated areas in Mymensingh city. The majority of the vendors were found making foods without formal training on food preparation and training and selling at open places such as riverside, parks, market area, roadside, etc.

### Sample collection and bacteriological analysis

A total of 40 samples of *phuchka* (*n* = 10), its egg toppings (*n* = 10), salad (*n* = 10), and vendors’ hand wash (*n* = 10) were collected aseptically in buffered peptone water in plastic ziplock bags and moved to the Department of Microbiology and Hygiene in Bangladesh Agricultural University ensuring a cool chain.

Exactly, 100 μl of 10-fold dilution from each sample was inoculated and spread aseptically onto a previously prepared plate count agar, Mannitol salt agar, and MacConkey agar (Hi-media, India) to determine total viable count (TVC), total coliform count (TCC), and total staphylococcal count (TSC), respectively [14]. The Gram-staining and biochemical tests such as sugar fermentation tests, indole production, methyl-red Voges–Proskauer, and motility tests were carried out to reveal the bacterial isolates’ identification.

### Antimicrobial susceptibility testing

The antimicrobial susceptibility of isolates was tested using the Kirby–Bauer disk diffusion method [15] on Mueller Hinton agar following the recommendations of the Clinical and Laboratory Standards Institute (CLSI) [16]. The isolated bacteria were grown in log phage, which was adjusted to 0.5 McFarland standard. The antibiotics tested in this study were ampicillin (10 μg), amoxicillin (30 μg), ciprofloxacin (5 μg), chloramphenicol (30 μg), gentamicin (10 μg), kanamycin (30 μg), penicillin G (10 U), nalidixic acid (30 μg), tetracycline (30 μg), and oxacillin (1 μg) for both *E. coli* and *Staphylococcus* spp. These antibiotics were chosen because they are commonly used to treat human diseases, particularly foodborne diseases. According to the CLSI zone diameter interpretive [16], antimicrobial testing results were classified as resistant or sensitive.

### Amplification of malB and tetA genes by polymerase chain reaction (PCR)

The list of primers with their sequences used for amplification of *malB* and tetracycline-resistant genes by PCR is listed in Table 1. The *tetA* gene was tested to understand the risk of acquiring tetracycline-resistant pathogen from *Phuchka*. The boiling method was used to extract the genomic DNA from bacterial isolates. To conduct the PCR, 5 μl DNA, 1 μl of both forward and reverse primers, 12.5 μl PCR master mixture (Promega, Madison, WI), and 5.5 μl nuclease-free water were mixed to make 25 μl of the reaction mixture. Initial denaturation at 95°C for 5 min was the first step of amplification, followed by denaturation at 94°C for 45 sec, extension at 72°C for 1 min, and the final extension ran for 5 min at 72°C. The entire reaction was repeated 30 times. The amplified PCR products were electrophoresed for 25 min at 100 volts in 1.5% agarose gel, then stained with ethidium bromide and seen under a ultraviolet transilluminator.

**Table 1.** PCR primers with sequences used in this study.

Genes	Primer sequences	Amplicon size (bp)	Annealing temperature	References
*malB-F*	5’GACCTCGGTTTAGTTCACAGA3’	585	58	[17]
*malB-R*	5’CACACGCTGACGCTGACCA3’			
*tetA-F*	5’GGTTCACTCGAACGACGTCA3’	577	54	[18]
*tetA-R*	5’CTGTCCGACAAGTTGCATGA3’			

## RESULTS

### Bacterial loads

The bacterial load results are put together and presented in Table 2. According to the results, all samples contained viable bacteria, with an average of 5.35 ± 0.59 log colony forming unit (CFU)/g, of which the highest average reading was found in the hand-wash samples with an average of 6.18 ± 1.70 log CFU/g. The presence of *Staphylococcus* spp. was observed in 82.5% of the samples. All the hand wash samples (100%) were found to be contaminated with *Staphylococcus* spp. (average 5.15 ± 1.22 log CFU/g) and the least contaminated samples were the egg toppings (70%) with an average of and 3.82 ± 1.50 log CFU/g. Coliform was found in 67.5% of the samples with a mean of 4.10 ± 0.73 log CFU/g. 40% samples of *phuchka* and egg toppings were contaminated with coliforms with an average of 4.56 ± 1.68 log CFU/g and 3.21 ± 1.00 CFU/g, respectively. Only 20% of the salad samples were contaminated with coliforms (3.70 ± 1.38 log CFU/g).

Based on the TVC value, 35% of the samples were found beyond satisfactory level according to the International Commission on Microbiological Specifications for Food (ICMSF) [19]. When TSC and TCC are taken into account, 42.5% and 62.5% of the samples were at an unacceptable level of contamination, respectively. All the mean values of TVC, TSC, and TCC of all samples were found at an unsatisfactory level.

### Antibiotic resistant pattern of the isolated bacteria

The data of antibiotic resistance pattern of Staphylococcus spp. and E. coli are summarized in Table 3. All the *Staphylococcus* spp. and *E. coli* isolates were ampicillin-resistant, and sensitive to ciprofloxacin and gentamicin. In the case of tetracycline, 58.33% *Staphylococcus* spp. and 41.66% *E. coli* were resistant to it. The pattern of tetracycline-resistant *E. coli* isolates was confirmed by the amplification of 577 bp size tetracycline-resistant *tetA* gene shown in Figure 2.

## Discussion

Most food hawkers in Bangladesh are unaware of food safety regulations and usually have no training. They prepare and store food without adhering to hygienic standards, exposing them to contamination from various sources such as raw foodstuffs, water, knives, utensils, flies, vendors’ bare hands, and occasional food handling by consumers [20,21]. Unhygienic conditions, such as sewage, an inefficient waste dumping system, and an insufficient water supply attract flies, contaminating food [22]. Our study shows results that are in line with the above comments.

According to the TVC value, 30% hand-wash samples and 70% *phuchka* and egg-topping samples were acceptable. On the contrary, other samples contained contamination of potential foodborne pathogens, such as *Staphylococcus aureus* and *E. coli*, more than the safety level. The overall TSC values in *phuchka* and its toppings varied from 40% to 80%. The TVC, TSC, and TCC values fluctuated from sample to sample. Out of 10 samples of each item, the 1st, 5th, and 6th samples were found to contain high levels of TVC, TSC, and TCC, indicating these samples were unsafe for human consumption. Handling food without using hand gloves can cause cross-contamination and, subsequently, transfer microbes into the food. It has been found in our survey study related to unhygienic conditions prevalent among street food vendors that 100% of the food vendors did not use aprons, while 98% handled with bare hands and 100% of the food vendors did not wear hair caps. Most of the vendors’ hand-wash water contained higher levels of contamination which might be due to repeated use of the same water or use of poor quality water for washing their hands and utensils. *Phuchka* and its toppings might be contaminated from the vendors’ hands during preparing or serving. Other reasons for contamination might due to keeping food for long periods without maintaining a temperature or pH or not using a spoon for serving food. The street food vendors brought food in their vending place at around 10 am and sold it up to the night, giving enough time for the organisms to grow.

**Table 2.** Total microbial load of TVC, TSC, and TCC in *Phuchka*, salad, egg toppings, and vendors’ hand wash.

Bacterial loads						
Sample name	TVC		TSC		TCC	
	No. (%)^a^	Mean ± SD^b^	No. (%)^a^	Mean ± SD	No. (%)^a^	Mean ± SD
*Phuchka* (*n* = 10)	10 (100%)	4.98 ± 1.16	8 (80%)	4.00 ± 1.31	4 (40%)	4.56 ± 1.68
Salad (*n* = 10)	10 (100%)	5.39 ± 2.04	8 (80%)	4.65 ± 1.66	2 (20%)	3.70 ± 1.38
Egg toppings (*n* = 10)	10 (100%)	4.85 ± 1.53	7 (70%)	3.82 ± 1.50	4 (40%)	3.21 ± 1.00
Hand wash (*n* = 10)	10 (100%)	6.18 ± 1.70	10 (100%)	5.15 ± 1.22	3 (30%)	4.75 ± 1.37
Total	40 (100%)	5.35 ± 0.59	33 (82.5%)	4.47 ± 0.61	27 (67.5)	4.10 ± 0.73

SD = Standard deviation.

^a^Number of positive samples.

^b^Mean bacterial count expressed in log CFU/g.

**Figure 1.**
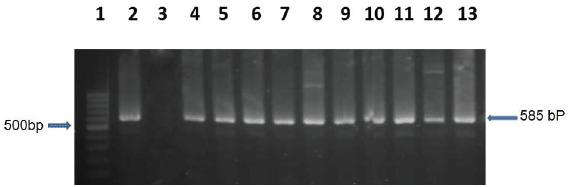
Amplified *malB* gene in *E. coli*; Lane 1: 100 bp DNA ladder; Lane 2: positive control; Lane 3: negative control; Lane 4–13: positive amplified genes.

**Figure 2.**
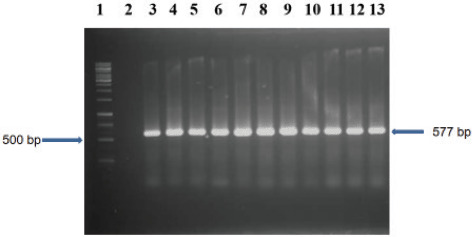
Amplified *tetA* gene in *E. coli*; Lane 1: 1 kbp DNA ladder; Lane 2: negative control; Lane 3–13: amplified 577 bp *tetA* gene in *E. coli* isolates.

Two different genera of bacteria, *Escherichia* and *Staphylococcus*, were identified in this study. Tambekar et al. [22] isolated *E. coli*, *S. aureus*, *Klebsiella* spp., and *Pseudomonas* spp. from street-vended food panipuri. Saxena and Agarwal [23] reported Shigella, *Salmonella*, *S. aureus*, *E. coli*, and *Bacillus cereus* in the street-vended bhelpuri and golgappa. 

In this study, both coagulase-positive and coagulase-negative *Staphylococci* were isolated and identified. When present in large numbers (more than 10^4^ CFU/g), the coagulase-positive *S. aureus* may cause foodborne illness [24]. Toxin formation in lethal amount by coagulase-positive *S. aureus* requires a large number (approximately 10^5^–10^6^ microorganisms per ml of food) [25]. *Staphylococcus* *epidermidis* is a natural flora found in human skin, respiratory system, urethra, external ear, and mouth, according to Nwamaka et al. [26]. Their appearance in the food samples was mostly attributable to the unsanitary behaviors of the food handlers.

*Escherichia coli*, confirmed by PCR (Fig. 1), may produce enterotoxins to cause foodborne diseases. The presence of *E. coli* in *phuchka* samples could be due to the insufficient cleanliness of food workers. Some of the likely explanations are the use of polluted water to wash salads and utensils, poor food storage at ambient temperatures in unsanitary places, facilities maintenance, and vendor personal hygiene [27].

Street foods carry multidrug-resistant bacteria, which may transfer to humans via the food chain [15]. In the current study, antibiotic resistance profiles of bacteria isolated from *phuchka* were also determined. Two genera of bacteria, *Escherichia* and *Staphylococcus*, were found to be resistant to three antibiotics. Antibiotic sensitivity test showed that all tested *Staphylococcus* spp. were 100% sensitive to ciprofloxacin and gentamicin, and least resistance (25%) was observed in the case of oxacillin. The oxacillin-resistant *S. aureus* isolates were also expected to be resistant to methicillin, which belongs to the same antibiotic class. *Staphylococcus aureus* that is resistant to methicillin (oxacillin) is known as methicillin-resistant *S. aureus*. Such organisms are also often found resistant to antimicrobials such as aminoglycoside, macrolide, chloramphenicol, tetracycline, and fluoroquinolones which are most commonly used [18]. On the other hand, *E. coli* demonstrated 100% resistance to ampicillin, followed by 66.66% to amoxicillin and 58.33% to tetracycline. The sensitivity results of *E. coli* showed 100% to ciprofloxacin and gentamicin, 91.66% to nalidixic acid, and 66.66% to chloramphenicol. The current study found that the most popular street food in Mymensingh, *phuchka*, was heavily contaminated with foodborne pathogens resistant to multiple antibiotics. Consuming these contaminated foods may result in foodborne infection and intoxication, making treatment with common antibiotics more difficult. 

**Table 3.** Antimicrobial resistance pattern of isolated *Staphylococcus* spp. and *E. coli* isolates.

Name of antibiotic disks	*Staphylococcus* spp.		*E. coli*	
	Sensitive (%)	Resistant (%)	Sensitive (%)	Resistant (%)
Amoxicillin	16.66	83.33	33.33	66.66
Ampicillin	00	100	00	100
Ciprofloxacin	100	00	100	00
Chloramphenicol	50	50	66.66	33.33
Gentamicin	100	00	100	00
Kanamycin	83.33	16.66	58.33	41.66
Nalidixic acid	58.33	41.66	91.66	8.33
Tetracycline	58.33	41.66	41.66	58.33
Penicillin G	33.33	66.66	–	–
Oxacillin	75	25	–	–

## Conclusion

The data from this study indicate that *phuchka* can pose a public health problem as multiple antibiotic-resistant foodborne bacteria are isolated from it. The most contaminated sources were the vendors’ hand washes. To reduce this contamination, vendors should be taught about maintaining personal hygiene. Further research regarding other foodborne pathogens can be detected on *phuchka*.

## List of Abbreviations

TVC: total viable count; TSC: total staphylococcal count; TCC: total coliform count; PCR: polymerase chain reaction; μl: microliter; CLSI: Clinical and Laboratory Standards Institute; DNA: deoxyribonucleic acid; °C: degree Celsius; CFU: colony forming unit.
